# Genetic parameter estimation for pre-weaning growth traits in Jordan Awassi sheep

**DOI:** 10.14202/vetworld.2018.254-258

**Published:** 2018-02-27

**Authors:** Khaleel Jawasreh, Zuhair Bani Ismail, Feizal Iya, Vielka Jeanethe Castañeda-Bustos, Mauricio Valencia-Posadas

**Affiliations:** 1 Department of Animal Production, Faculty of Agriculture, Jordan University of Science and Technology, Jordan; 2 Department of Veterinary Clinical Sciences, Faculty of Veterinary Medicine, Jordan University of Science and Technology, Jordan; 3 Department of Genetics and Biostatistics, Faculty of Veterinary Medicine and Zootechnics, National Autonomous University of Mexico, Ciudad Universitaria, CDMX 04510, Mexico; 4 Department of Veterinary and Zootechnics, Life Sciences Division, Campus Irapuato-Salamanca, University of Guanajuato, Ex Hacienda El Copal, Irapuato, Guanajuato 36500, Mexico

**Keywords:** Awassi sheep, breeding values, growth traits, heritability

## Abstract

**Aim:**

The aim of this study was to estimate the heritability, genetic and phenotypic correlations, and the genetic trends for pre-weaning growth traits including the birth weight (BWT), weaning weight (WWT), pre-weaning daily gain (PWDG), and weaning age (WA) in Awassi lambs.

**Materials and Methods:**

A total of 5131 Awassi lambs from two Jordanian sheep breeding stations were used. A multitrait animal model and restricted maximum likelihood methods were used to estimate the covariances between the studied traits.

**Results:**

The mean±standard deviation of BWT, WWT, PWDG, and WA was 4.48±0.8 kg, 17.13±0.7 kg, 0.2±0.07 g, and 65.5±0.7 days, respectively. Heritability estimates were 0.30±0.04 for BWT, 0.19±0.04 for WWT and PWDG, and 0.2±0.04 for WA. Positive genetic correlations were obtained between BWT and other traits, while negative correlations were obtained between WWT, PWDG, and WA (−0.50±0.12) and between WWT and WA (−0.67±0.14). The positive phenotypic correlation was obtained between WA and PWDG (0.63±0.01). The highest additive genetic variance was obtained for WA (34.58), while the lowest was estimated for PWDG (6.22E-04). The highest phenotypic variance was obtained for WA (175.5), while the lowest value obtained was for BWT (0.54). Maternal additive variance ranged between 0.13 and 0.03. The genetic trends were around zero for all studied traits.

**Conclusion:**

Selection should be conducted using animals with high estimated breeding values through controlled breeding.

## Introduction

Genetic selection is one of the most important methods that has been used to maximize the expected genetic gain of livestock through crossbreeding programs and breed selection using specific gene markers or genomic selection methods [1]. Awassi sheep is the most common breed of sheep in Jordan. Awassi breed is considered as an important genetic resource that plays a significant role in sheep industry in more than 30 countries in the Middle East as well as worldwide [2]. Many non-genetic factors are known to affect different quantitative traits of lambs [3]. It has been reported that sex, genetic group, and season of birth have a significant effect on the traits such as birth weight (BWT), weaning age (WA), and weaning weight (WWT) [1-3].

Heritability, genetic correlation, and breeding values are considered important factors that determine the lamb’s genetic gain and therefore performance and economic value of the lamb [4,5]. Studying the genetic variance of important traits and covariance between these traits is considered an important step in the planning and implementation of any successful selection or breeding program that aims to improve the genetic gain of animals.

In Jordan, phenotypic selection of sheep has been applied in two breeding stations using the WWT as the sole selection criteria. To the best of our knowledge, the present investigation is the first one that studies the effects of various maternal and environmental factors on the growth traits of Awassi sheep in Jordan. Therefore, the aim of this study was to estimate the genetic and phenotypic parameters and genetic trends of the BWT, WWT, pre-weaning daily gain (PWDG), and WA in Awassi lambs.

## Materials and Methods

### Ethical approval

This study was reviewed and all experimental procedures were approved by the Jordan University of Science and Technology Animal Use and Care Committee (JUST-ACUC).

### Location

The study was conducted at two major Jordanian sheep breeding stations (AL-Khanasry and Al-Fjaj stations) that belong to the Ministry of Agriculture. AL-Khanasry Research station is located about 65 km to the Northern east of Amman, at 32°30ʹ N, and an altitude of 860 m above sea level. The annual total rain precipitation ranges between 150 and 200 mm [6]. Al-Fjaj station is located in the Southern part of Jordan, about 210 km to the South of Amman, at an altitude of 1800 m above sea level, and annual rainfall of about 110 mm.

### Flock management

Flocks are managed using a semi-intensive system. Grazing is allowed for 4 h in the morning and 3 h in the afternoon year-round, except during pregnancy period. Rangeland consisted of green fodder and natural vegetation (shrubs and herbage) mostly from mid-January to late April. Each ewe receives 0.5 kg of alfalfa and 1.5 kg of concentrate during the past 8 weeks of gestation. After lambing, the amount of concentrate offered increases to 1.8 kg. Concentrate is composed of barley (67.5%), soybean meal (12%), wheat bran (18%), limestone (1.4), salt (1%), and trace minerals (0.1) in both stations.

During the breeding season, group mating strategy is applied in the two stations. Before lambing, each ewe is placed in an individual pen that allows the ewe to take care of her newborn and stays this pen for about 3 days. Through this period, the ewe and her lamb are weighed and the lamb is identified with a permanent ear tag. All information is recorded for the newborn (sex, type of birth [single or twin], date of birth, dam ID, sire ID, and BWT).

Each lamb is allowed to consume the recommended amount of colostrum immediately after lambing. The regular lactation period starts and continues when the lamb is allowed to suckle freely from his dam during the full day for up to 15 days. Creep feeding starts at 15 days of age. Lambs are weighted biweekly to monitor their body weights and to prepare them for weaning. Weaning strategy for both stations is based on lamb weight and age. Lambs are weaned when they reach 14 kg of body weight. On the day of weaning, the lamb’s weight and age are recorded.

### Data collection

The data were collected through 13 years (1999-2011) from AL-Khanasry and AL-Fjaj stations. The same traits and measurements were taken using similar procedures in the two stations.

### Statistical and genetic analysis

Data were analyzed with a linear mixed model using average information- restricted maximum likelihood methodology. To estimate the variance of BWT, WWT, PWDG, and WA, a multitrait animal model was used. The fixed part of the models for analysis of the traits included birth type (single or twin), lamb’s sex, and location. The fixed effect of year was considered for WWT, PWDG, and WA. The model used the BWT as a covariate. Animal effect, maternal effect, and residual effects were included as random effects. The model used for each trait, expressed in matrix notation [7], was as follows:

Y=Xb+Zu+Wm+e

Where Y=Vector of observations for each traits; X, Z, and W are the incidence matrices of fixed, additive genetic effect of the animal, and maternal permanent environment effect, respectively; b=Vector of fixed effects for the traits analyzed; u=Vector of random effects of the additive genetic effect of the animal; m=Vector of the permanent environmental effect; e=Vector of random error.

Genetic correlations were obtained through bivariate analysis including the same effects included in the univariate model mentioned above. The analysis was performed using the software ASReml 3.0 [7].


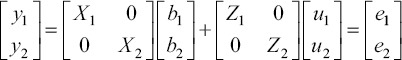


### Genetic and phenotypic trends

Genetic trend for the traits was calculated by estimating the predicted breeding values on the year of birth. Phenotypic trend also calculated using the regression of phenotypic values of BWT, WWT, and PWDG on the year of birth.

## Results

### Descriptive statistics

The descriptive statistics and probability values of the studied traits are presented in Tables-1 and 2. The sex of the lamb appeared to significantly affect all studied pre-weaning traits (p<0.001). The effect of sex on BWT was observed to be consistent. Males were heavier (4.4±0.02 kg) at birth than females (4.2±0.01 kg). WWT was also affected significantly sex (p<0.001) where females were lighter than males. Males grew significantly faster (PWDG=0.19 g/day; p<0.001) than females (PWDG=0.17 g/day). Sex was also found to have a significant effect (p<0.001) on WA. Males were weaned at 64.4± 0.39 days, while females were weaned at 65.6±0.37 days.

**Table-1:** Descriptive statistics for birth weight, weaning weight, weaning age, and pre-weaning daily gain in Awassi lambs.

Variable	N	Mean±SD	CV
Birth weight (kg)	3812	4.48±0.8	18
Weaning age (days)	3251	65.40±0.7	25
PWDG (kg)	3251	0.20±0.07	32
Weaning weight (kg)	3251	17.13±0.7	25

N=Number of samples, SD=Standard deviation, CV=Coefficient of variability, PWDG=Preweaning daily gain

**Table-2:** Probability values (p values) of nongenetic factors effects on pre-weaning growth traits of Awassi lambs.

Fixed effect	Birth weight	Weaning weight	Pre-Weaning daily gain	Weaning age
Sex	<0.0001	<0.0001	0.0053	0.0084
Type of birth	<0.0001	<0.0001	<0.0001	<0.0001
Geographic location	<0.0001	<0.0001	<0.0001	<0.0001
Year of birth	<0.0001	<0.0001	<0.0001	<0.0001
Birth weight	-	<0.0001	<0.0001	<0.0001

The effect of type of birth (single vs. twins) on BWT, WWT, PWDG, and WA was highly significant (p<0.001).

The BWT, WWT, PWDG, and WA were affected significantly by geographical location. Lambs born in AL-Khanasry station were heavier (p<0.001) at birth and at weaning than lambs born in AL-Fjaj station. The PWDG of lambs in AL-Khanasry station was also significantly more than that of in AL-Fjaj. At Al-Khanasry station, lambs were weaned significantly earlier than those in Al-Fjaj.

The year of birth was also found to have a significant effect (p<0.001) on all pre-weaning growth traits. The maximum BWT average was in 2006 (4.43±0.037 kg), while the lowest weight was in 2009 (4.14±0.068 kg). The WWT was highest (p<0.01) in 2003 (20.61±0.26 kg), while the lowest (p<0.01) WWT was in 2008 (15.82±0.19 kg). The highest PWDG was in 2003 (0.23±0.006 g/day), while the lowest was in 2008 (0.13±0.0043 g/day). The year of birth had a significant effect (p<0.05) on WA. In 2008, the WA was 79.54±0.76 days, while in 2003, the WA was 53.74±1.02 day.

### Genetic parameters

The additive genetic variance (σ^2^a), phenotypic variance (σ^2^p), and maternal genetic effect (σ^2^mg) of pre-weaning growth traits in Awassi lambs are presented in Table-3. The highest additive genetic variance was obtained for WA (34.58), while the lowest was estimated for PWDG (6.22E-04). The additive genetic variance (σ^2^ a) of growth traits was 0.17, 2.24, 6.22E-04, and 34.58 for BWT, WWT, PWDG, and WA, respectively. The highest phenotypic variance was estimated for WA and the lowest was observed for PWDG. Phenotypic variances (σ^2^ p) were 0.54, 12.0, 3.34E-03, and 175.5 for BWT, WWT, PWDG, and WA, respectively. The maternal genetic effect on BWT was 0.13, 0.06 on WWT and ADG, and 0.03 on WA.

**Table-3:** Additive genetic variance (σ^2^a), phenotypic variance (σ^2^p), and maternal genetic effect (σ^2^mg) of growth traits studied in Awassi lambs.

Trait	Additive genetic variance (σ^2^a)	Phenotypic variance (σ^2^p)	Maternal genetic effect (σ^2^mg)
Birth weight	0.17	0.54	0.13±0.02
Weaning weight	2.24	12.0	0.06±0.02
Pre-Weaning daily gain	6.22E04	3.34E03	0.06±0.02
Weaning	34.58	175.5	0.03±0.02

### Heritability, genetic and phenotypic correlations

The heritability, genetic and phenotypic correlations among pre-weaning growth traits in Awassi lambs are presented in Table-4. The estimated heritability of BWT, WWT, PWDG, and WA was 0.30±0.04, 0.19±0.04, 0.19±0.04, and 0.20±0.04, respectively. Genetic correlations between BWT and the other traits ranged from 0.61 (PWDG) to 0.65 (WA). The phenotypic correlations between BWT and the other trait ranged from 0.47 (PWDG) to 0.63 (WA). The genetic and phenotypic correlations between WWT and PWDG were positive, while for the WWT and WA, were found negative correlations. The genetic and phenotypic correlations between PWDG and WA were also negative.

**Table-4:** Heritability (values in diagonal direction), genetic (values above diagonal), and phenotypic (values below diagonal) correlations between pre-weaning growth traits in Awassi lambs.

Traits	Birth weight	Weaning weight	Pre-Weaning daily gain	Weaning age
Birth weight	0.30±0.04	0.63±0.08*	0.62±0.08*	0.65±0.07*
Weaning weight	0.48±0.02*	0.19±0.04	0.67±0.14*	−0.50±0.1*
Pre-Weaning daily gain	0.48±0.02*	0.85±0.005*	0.19±0.04	−0.50±0.12*
Weaning age	0.63±0.01*	−0.44±0.02*	−0.44±0.02*	0.20±0.04

* p<0.05

### Genetic and phenotypic trends

Genetic trends for the studied traits are presented in Table-5. The genetic trend for BWT was positive and highly significant (p<0.001) in both stations. At Al-Khanasry station, the genetic trend for WWT and PWDG was negative, while at AL-Fjaj station, it was positive for both mentioned traits.

**Table-5:** Genetic trend for pre-weaning growth traits in Awassi lambs.

Traits	All population	AlKhanasry station	AlFjaj station
Birth weight	−0.0005±0.0006**	0.004±0.0009**	−0.006±0.0008**
Weaning weight	0.006±0.002**	−0.005±0.003**	0.03±0.002**
Pre-Weaning daily gain	0.0001±0.00003**	−0.0001±0.00005**	0.0005±0.00004**

** p<0.001

## Discussion

### Fixed effects

In the present report, similar to the previous ones, the fixed effect of lamb’s sex on growth performance was found to have a significant effect [8-10]. The effect of sex on BWT could be explained by differences in hormonal effects, number of cotyledons (higher in ewes carrying male lambs), and placental weight (heavier in ewes carrying male lambs) [10].

In this study, there was a significant difference in various pre-weaning growth parameters between two different geographically located breeding stations in Jordan. Significant variations of body weights between different farms may reflect the level of feeding programs, general farm management, and environmental conditions under which the flocks are maintained. Similar results have been reported previously by many authors [11,12]. Another aspect that has to be considered concerning pre-weaning growth traits is competition between twins for consuming milk from their dams which explains the slow growth rate of the twins compared to the singles [13].

### Genetic and phenotypic parameters and maternal effects

Animal growth rate is influenced not only by direct additive genetic effects but also by parental genetic effects [5]. Additive genetic effect is the part of genes that can be transmitted from parents to offspring [4]. The maternal genetic effects are expressed during gestation and lactation and directly affect the lamb’s BWT. This effect, however, usually diminishes slowly as the lamb becomes older [14]. In this study, the additive genetic variance estimated for BWT was higher than that reported previously in Bharat Merino sheep [15] and for Menz sheep [16] and was lower than the estimated values by other authors [17,18]. Differences in genetic additive effects on lamb’s pre-weaning growth traits could be related to differences in farm management, different sheep breeds, and various environmental factors.

In this study, the results of phenotypic variance for WWT were similar to those reported in Kurdish sheep [19]. However, the higher phenotypic variance was estimated in Dormer sheep, Ile de France, Merino land sheep, and Arabi lambs [18,20]. Lower phenotypic variance for WWT was reported in Kermani and Menz sheep [13,16].

The phenotypic variance for WA reported in Dormer sheep and Kurdish sheep was lower than the results obtained in this study [18,19]. The phenotypic variance estimate for PWDG in Awassi lambs was lower than the reports in Kurdish and Arabi lambs [13,19].

The estimated maternal variance for BWT reported in this study in Awassi lambs is higher than that reported in Dormer Kurdish and Malpura sheep [19-21], while similar results were obtained in Ile de France and Malpura sheep [15,20]. Nevertheless, higher values have been reported by other authors in Kermani and Moghani sheep [13,17]. Similar results concerning the estimated maternal variance for PWDG were reported previously [15,22].

### Heritability estimates

In general, the heritability estimates for various pre-weaning growth traits in this study were low to medium. Interestingly, the heritability estimates appeared to decrease gradually over age. The declining heritability over age of the lamb can be explained by poor environmental conditions or poor management in the postpartum period [4]. Similar results were also reported previously in Arman and Kurdish sheep [19,23]. In this study, higher heritability values for WWT and PWDG than values reported in Suffolk lambs [24]. However, the heritability estimates for BWT were higher than that obtained herein Awassi lambs [24]. On the other hand, higher estimates of heritability for WWT were reported by many researchers in different breeds [13,14,16].

### Genetic and phenotypic correlations and genetic trends

The genetic correlation between any two traits may be attributed to the pleiotropic effect, while phenotypic correlations are explained by the common environmental effects that different traits may share [4]. The genetic correlations between various pre-weaning growth traits in this study were higher than the phenotypic correlations. Similar results were reported previously in South African D’man ewes and Sabi sheep of Zimbabwe [22]. In this study, the results of genetic trends for various pre-weaning growth traits were similar to those reported in Merino sheep, Kermani sheep, and Zel sheep [24,25].

## Conclusion

Results of this study indicate that selection should be conducted using animals with high estimated breeding values through controlled breeding.

## Authors’ Contributions

KJ: Designed the experiment, organized the experimental work, and performed the statistical analysis. ZBI: Scientific and language editing, manuscript writing. FI: Drafted the manuscript, conducted field visits and data collection and interpretation. VJC: Performed the genetic analysis. MVP: Read and revised the manuscript. All authors read and approved the final manuscript.

## References

[1] Gondro C, van der Werf J, Hayes B (2013). Genome wide association studies and genomic prediction. Methods in Molecular Biology.

[2] Galal S, Gursoy O, Shaat I (2008). Awassi sheep as a genetic resource and efforts for their genetic improvement. Small Rum. Res.

[3] Yilmaz O, Denk H, Bayram D (2007). Effects of lambing season, sex and birth type on growth performance in Norduz lambs. Small Rum. Res.

[4] Falconer D.S, Mackay T.F.C (1996). Introduction to Quantitative Genetics.

[5] Jalil-Sarghale A, Kholghi M, Moradi S.M, Moradi S.H, Mohammadi H, Abdollahi-Arpanahir R (2014). Model comparisons and genetic parameter estimates of growth traits in Baluchi sheep. Slovak. J. Anim. Sci.

[6] M.O.A (2012). Ministry of Agriculture, Statistical Year Book, Kingdom of Jordan.

[7] Gilmour A.R, Gogel B.J, Cullis B.R, Welham S.J, Thompson R (2007). ASReml user Guide Release 2.0. VSN International Ltd. 5 the Water's House, Water House Street Hemel, Hempstead.

[8] Gootwine E, Rozov A (2006). Seasonal effects on birth weight of lambs born to prolific ewes maintained under intensive management. Livest. Sci.

[9] Jawasreh K.I.Z, Khasawneh A.Z (2007). Studies of some economic characteristic on Awassi lambs in Jordan. Egyptian J Sheep Goat Sci.

[10] Jawasreh K.I. Z, Awawdeh F.T, Al-Khasawneh A.Z, Shdaifat B, Al-Shboul H, Al-Hamed B (2009). The effect of some placental factors in birth weight of Awassi lambs. Res. J. Anim. Vet. Sci.

[11] Oosting S.J, Mekoya A, Fernandez-Rivera S, Van der Zijpp A.J (2011). *Sesbania sesban*as a fodder tree in Ethiopia livestock farming systems feeding practices and farmers perception of feeding effects on sheep performance. Livest. Sci.

[12] Supakorn C, Pralomkarn W, Anothaisinthawee S (2013). Estimation of genetic parameters and genetic trends for weight and body measurements at birth in sheep populations in Thailand. Songklanakarin J. Sci. Technol.

[13] Rashidi A, Mokhtari M.S, Safi J.A, Mohammad A.M.R (2008). Genetic parameter estimates of pre-weaning growth traits in Kermani sheep. Small Rum. Res.

[14] Prakash V, Prince L.L.L, Gowane G.R, Arora A.L (2012). The estimation of (co) variance components and genetic parameters for growth traits and Kleiber ratios in Malpura sheep of India. Small Rum. Res.

[15] Gowane G.R, Chopra A, Prakash V, Arora A.L (2010). Estimates of (co)variance components and genetic parameters for body weights and first greasy fleece weight in Malpura sheep. Livest. Sci.

[16] Gizaw S, Lemma S, Komen H, Johan A.M (2007). Estimates of genetic parameters and genetic trends for live weight and fleece traits in Menz sheep. Small Rum. Res.

[17] Jafaroghli M, Rashidi B, Mokhtari M.S, Shadparvar A.A (2010). (Co) variance components and genetic parameter estimates for growth traits in Moghani sheep. Small Rum. Res.

[18] Roshanfekr H, Mamouei M, Mohammadi K, Rahmatnejad E (2011). Estimation of genetic and environmental parameters affected pre-weaning traits of Arabi lambs. J. Anim. Vet. Sci.

[19] Borhan S.H, Masoud Z (2012). Estimation of genetic parameters for body weights of Kurdish sheep in various ages using multivariate animal models. Afr. J. Biotechnol.

[20] Zishiri O.T, Cloete S.W.P, Olivier J.J, Dzama K (2014). Genetic parameters for live weight traits in South African terminal sire sheep breeds. Small Rum. Res.

[21] Mishra A.K, Arora A.L, Prince L.L.L, Kumar S, Gowane G.R (2009). Genetic analysis of performance traits of Malpura sheep. Indian Vet. J.

[22] Abbasi M.A, Abdollahi-Arpanahi R, Maghsoudi A, Vaez T.R, Nejati-Javaremi A (2012). Evaluation of models for estimation of genetic parameters and maternal effects for early growth traits of Iranian Baluchi sheep. Small Rum. Res.

[23] Mokhtari M.S, Moradi S.M, Moradi S.H, Sadeghi M (2013). Estimation of (co) variance components and genetic parameters for growth traits in Arman sheep. J. Livest. Sci. Technol.

[24] Mokhtari M.S, Rashidi A (2010). Genetic trends estimation for body weight of Kermani sheep at different ages using multivariate animal models. Small Rum. Res.

[25] Mohammadi H, Sadeghi M (2011). Estimates of genetic and phenotypic trends for body weight traits of Zel sheep obtained by univariate and multivariate animal model analysis. J. Anim. Sci.

